# Taxa‐dependent temporal trends in the abundance and size of sea urchins in subtropical eastern Australia

**DOI:** 10.1002/ece3.11412

**Published:** 2024-05-19

**Authors:** Emily McLaren, Brigitte Sommer, John M. Pandolfi, Maria Beger, Maria Byrne

**Affiliations:** ^1^ School of Life and Environmental Sciences, Coastal and Marine Ecosystems Group The University of Sydney Sydney New South Wales Australia; ^2^ School of the Environment The University of Queensland St. Lucia Queensland Australia; ^3^ School of Biology, Faculty of Biological Sciences University of Leeds Leeds UK; ^4^ Centre for Biodiversity and Conservation Science, School of the Environment The University of Queensland St. Lucia Queensland Australia

**Keywords:** echinoids, New South Wales, population dynamics, sea urchins, subtropical reefs, transition zone

## Abstract

Subtropical reefs host a dynamic mix of tropical, subtropical, and temperate species that is changing due to shifts in the abundance and distribution of species in response to ocean warming. In these transitional communities, biogeographic affinity is expected to predict changes in species composition, with projected increases of tropical species and declines in cool‐affinity temperate species. Understanding population dynamics of species along biogeographic transition zones is critical, especially for habitat engineers such as sea urchins that can facilitate ecosystem shifts through grazing. We investigated the population dynamics of sea urchins on coral‐associated subtropical reefs at 7 sites in eastern Australia (28.196° S to 30.95° S) over 9 years (2010–2019), a period impacted by warming and heatwaves. Specifically, we investigated the density and population size structure of taxa with temperate (*Centrostephanus rodgersii, Phyllacanthus parvispinus*), subtropical (*Tripneustes australiae*) and tropical (*Diadema* spp.) affinities. Counter to expectation, biogeographic affinity did not explain shifts in species abundances in this region. Although we expected the abundance of tropical species to increase at their cold range boundaries, tropical *Diadema* species declined across all sites. The subtropical *T. australiae* also showed declines, while populations of the temperate *C. rodgersii* were remarkably stable throughout our study period. Our results show that temporal patterns of sea urchin populations in this region cannot be predicted by bio‐geographic affinity alone and contribute critical information about the population dynamics of these important herbivores along this biogeographic transition zone.

## Graphical abstract

We investigated the population dynamics of sea urchins on coral‐associated subtropical reefs at 7 sites in eastern Australia (28.196° S to 30.95° S) over 9 years (2010–2019), a period impacted by warming and heatwaves. Counter to expectation, biogeographic affinity did not explain shifts in species abundances in this region. Our results show that temporal patterns of sea urchin populations in this region cannot be predicted by bio‐geographic affinity alone and contribute critical information about the population dynamics of these important herbivores along this biogeographic transition zone.

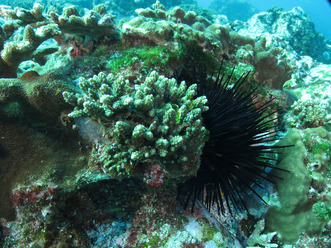

## 1 INTRODUCTION

Marine biogeographic transition zones represent a gradient between tropical and temperate ecosystems (Horta e Costa et al., 2014) and feature a mix of tropical, subtropical and temperate species arrayed along gradients of environmental conditions (Beger et al., 2014; Sommer et al., 2014). These transition zones are often influenced by western boundary currents that transport propagules of tropical and subtropical species poleward, as seen in Australia and Japan (Vergés et al., 2014). In these regions, many species live at the edges of their warm or cool thermal ranges, making them highly vulnerable to climate driven thermal anomalies (Beger et al., 2014; Mulders et al., 2022; Poloczanska et al., 2016; Stuart‐Smith et al., 2022; Zarzyczny et al., 2023).

Globally, there has been a shift from the dominance of cool to warm affinity species in marine transition zones, a phenomenon called topicalization (Mulders et al., 2022; Vergés et al., 2014). In a warming ocean, range shifts are often explained by biogeographic affinity, with warm‐affinity species increasing in abundance and diversity at their leading edges and cold‐affinity species decreasing at their warm‐range margins (Horta e Costa et al., 2014; Mulders et al., 2022; Poloczanska et al., 2016; Stuart‐Smith et al., 2022; Zarzyczny et al., 2023). Along with the direct effects of heat stress on temperate species, such as cold‐water kelps, topicalization is often characterised by changes in biotic interactions, including marked changes in herbivory due to the range extension and increased abundance of tropical herbivorous fishes (Vergés et al., 2014, 2016; Wernberg et al., 2016).

Herbivory is amongst the most important ecological interactions in marine ecosystems (Bernes et al., 2018; Côté et al., 2004; Futuyma & Agrawal, 2009; Hawkes & Sullivan, 2001; Milchunas & Lauenroth, 1993; Naeem et al., 2016; Ohgushi, 2005). In particular, sea urchins are important habitat engineers and can play a pivotal role in mediating benthic dynamics in warming seas (Lessios, 2016; Lessios et al., 2001; Ling, 2008; Ling et al., 2018; Zarzyczny et al., 2022). A long history of exclusion experiments and die‐off events show the ecological role of sea urchin herbivory in marine ecosystems (Steneck, 2020; Sweet, 2020). For example, in the Caribbean, herbivory by the diadematid sea urchin *Diadema antillarum*, was key in maintaining the health of coral reefs by controlling turfing algae until its die‐off from an unknown pathogen precipitated a phase shift from a coral dominated to a turf dominated ecosystem in the 1980s (Hughes et al., 2007; Lessios, 2016; Lessios et al., 2001). In temperate ecosystems, high sea urchin abundance can result in phase shifts from macroalgae dominated to sea urchin and coralline algae dominated systems, termed ‘barrens’ (Eklöf et al., 2008; Filbee‐Dexter & Scheibling, 2014; Ling, 2008; Ling et al., 2019; Pederson & Johnson, 2008; Steneck et al., 2002). Although sea urchins can also be abundant along subtropical transition zones, where they are hypothesised to accelerate topicalization through grazing (Schuster et al., 2022), the population ecology of sea urchin species with different thermal affinities is not well understood.

On the east coast of Australia, the biogeographic transition zone is centred around the Solitary Islands Marine Park (SIMP, 30° S), which hosts a high diversity of tropical, subtropical and temperate species, including endemic species and corals at their poleward range limits (Baird et al., 2017; Ferrari et al., 2018; Malcolm et al., 2007, 2010; Malcolm & Ferrari, 2019; Mizerek et al., 2021; Smith & Peregrin, 2020; Sommer et al., 2014, 2017). The ecological dynamics of this region are influenced by the East Australian Current (EAC), and south‐eastern Australia is considered a global warming hot spot where increasing flow of the EAC is driving the poleward range extensions of many marine species (Hobday & Pecl, 2014; Ridgway & Godfrey, 1997; Suthers et al., 2011; Vergés et al., 2014, 2016).

Ecological dynamics along this biogeographic transition zone vary among taxa and are mediated by latitudinal and seasonal variation in temperature, ongoing ocean warming and heatwave anomalies (Bates et al., 2014; Lachs et al., 2021; Malcolm et al., 2011; Sommer et al., 2018). For example, patterns in coral community composition are distinct from those occurring in the Great Barrier Reef (Sommer et al., 2014, 2017), and the abundance of tropical corals has remained remarkably stable since the 1990s (Mizerek et al., 2021). However, in 2016, a significant heatwave resulted in the mass bleaching of coral, including the endemic subtropical coral *Pocillopora aliciae* resulting in high mortality with limited recovery of this species in subsequent years (Kim et al., 2019; Lachs et al., 2021). Recently, kelp cover in the Solitary Islands region has declined in response to warming in parallel with an increase in tropical fishes, including tropical herbivorous fishes (Smith et al., 2021; Vergés et al., 2016).

While there have been extensive investigations of the population dynamics of corals and fishes in the east Australian transition zone with respect to regional warming (Cant et al., 2021, 2023; Lachs et al., 2021; Malcolm et al., 2010; Malcolm & Ferrari, 2019; Sommer et al., 2014, 2018; Vergés et al., 2016), less is known about sea urchins and other benthic species. The subtropical reefs around the SIMP have high diversity of sea urchins, with 26 species recorded to date (Shaw, 2023), including tropical (e.g. *Diadema*, *Pseudoboletia*, and *Tripneustes*) and temperate (*Heliocidaris*, *Centrostephanus*) species. The abundant *Tripneustes* species in this region and in more temperate latitudes was previously considered to be a range extension of the tropical species *T. g. gratilla* (Castro et al., 2020), until recent taxonomic studies showed that this species, *Tripneustes australiae*, is a subtropical species endemic to south‐east Australia and the west coast of New Zealand (Bronstein et al., 2019; McLaren et al., 2023).

Ocean warming and changes to the EAC have driven the poleward range expansion of *Centrostephanus rodgersii* from New South Wales (NSW) to Tasmania, with major impacts on receiving ecosystems resulting in the shift from macroalgal reefs to ‘barrens’ habitats (Byrne & Andrew, 2020; Ling, 2008). Within its native distribution in NSW, *C. rodgersii* contributes to the formation of a mosaic of ‘barren’ and kelp forest habitats characteristic of the region and is important for local biodiversity (Curley et al., 2002; Kingsford & Byrne, 2023; Underwood et al., 1991). Understanding spatial and temporal dynamics of common sea urchin species including *C. rodgersii* on subtropical reefs in NSW is critical to understanding future trajectories of associated ecosystems.

Here we investigated the population dynamics of sea urchins on subtropical reefs of east Australia over a nine‐year period of warming and heatwaves that impacted coral, kelp, and fish assemblages in the region (Kim et al., 2019; Malcolm & Ferrari, 2019; Vergés et al., 2016). We quantified the abundance and population size structure of four sea urchin species to assess population dynamics and potential recruitment and mortality (Anderson & Pratchett, 2014; Ebert et al., 2019; Meesters et al., 2001). As the region has been warming for decades (Malcolm & Ferrari, 2019; Vergés et al., 2016), we also aimed to understand the potential influence of temperature on sea urchin population dynamics. Overall, we hypothesised that biogeographic affinity predicts patterns in sea urchin populations over time and warm affinity/tropical species would increase throughout the duration of the study, while cool tolerant/temperate species would decrease.

## 2 METHODS

### 2.1 Study region and species

**FIGURE 1:**
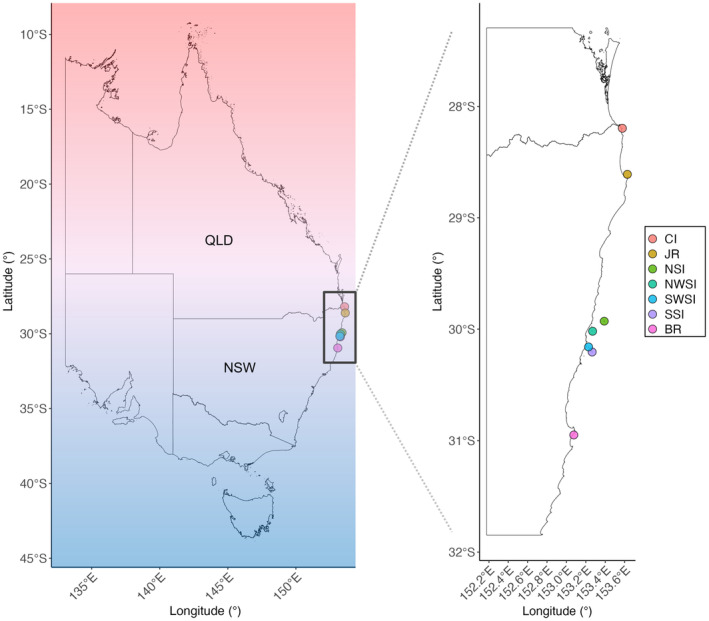
Location of the seven study sites across the biogeographic transition zone (28.196° S to 30.95° S) of eastern Australia: Cook Island (CI), Julian Rocks (JR), North Solitary Island (NSI), Northwest Solitary Island (NWSI), Southwest Solitary Island (SWSI), South Solitary Island (SSI) and Black Rock (BR). Red to blue gradient represents the thermal gradient along the east coast of Australia from tropical to temperate thermal regimes.

This study determined the temporal and thermal pattens in the abundance and size structure of sea urchin populations in subtropical New South Wales (NSW), east Australia. We measured the size and densities of all sea urchin species encountered in surveys from 2010 to 2019 across 7 sites (Figure 1) spanning a latitudinal gradient along the subtropical‐to‐temperate transition zone (28.196° S to 30.95° S). We also recorded whether the sea urchin species were tropical, subtropical or temperate (Table S1).

### 2.2 Sea urchin surveys and image analysis

Sea urchin surveys in coral associated habitat were conducted at the study sites (Figure 1) during August to October in 2010, 2012, 2016 and 2019; except Julian Rocks which was sampled in March 2016, and South Solitary Island which was sampled in March 2011. Black Rock could not be accessed in 2012. At each sampling time, five replicate 30 m long belt transects were laid at least 10 m apart and downward facing photographs were taken at each meter interval. Each photograph captured a 1 m^2^ area, resulting in a 30 by 1 m^2^ belt transect (see Sommer et al., 2014) (Figure S1). All transects were laid randomly in the same habitat at each site at each sampling timepoint.

The sea urchins observed in the 1 m^2^ images were identified to species where possible, counted and outlined in ImageJ to determine test diameter. Urchins were only outlined if more than 50% of their body was present in the image so that an accurate diameter could be extracted. Each sea urchin was outlined using the SizeExtractR workflow in ImageJ, and data were extracted using the SizeExtractR R package (Lachs et al., 2022). Ferets' diameter, or the maximum calliper length, which is the longest distance between the outlined boundaries, was determined for each sea urchin. We were unable to consistently distinguish between *D. setosum* or *savignyi* from the images and both could possibly occupy the study sites, so these two species were grouped as *Diadema* spp.

### 2.3 Sea surface temperature analysis

Monthly average sea surface temperature (SST) data were extracted for all 7 sites from the Modis Aqua satellite (Parkinson, 2003) at 1 km spatial resolution for each of the seven sites for 2003–2019 (Figure S2, Table S2). From these data, we calculated the annual mean, minimum and maximum temperatures (±SEM) (°C), temperature ranges for each site and year, and the mean annual mean, minimum and maximum temperature across all years and sites (Tables S3 and S4).

### 2.4 Temporal patterns in the size structure of sea urchin populations

Counts and test diameter (i.e. the diameter at the widest point of the sea urchin skeleton in the photograph, not including spines) data were used to determine the size frequency distribution and density of sea urchins (m^−2^) in 2010/11, 2016 and 2019 and to determine the temporal dynamics of the populations. These data were used to infer population size structure through time. For 2012, only counts and not measures of test diameter were conducted and population size structure analyses therefore do not include data for the year 2012.

### 2.5 Statistical analysis

#### 2.5.1 Thermal and spatial trends in sea urchin densities

All statistical analyses were performed in R (R Core Team, 2021). Of the 8 species observed in the transects, only *Centrostephanus rodgersii, Tripneustes australiae*, *Diadema* spp. and *Phyllacanthus parvispinus* were sufficiently abundant for species analysis. To test if the density of these species (*C. rodgersii, T. australiae*, *P. parvispinus* and *Diadema* spp.) varied with thermal conditions we used separate Generalised Linear Mixed Model (GLMM) with a Poisson distribution and a log link function for each species, with mean annual temperature (for each site) as a fixed effect and the random effects of site and transect (transect nested in site) (*n* = 5) in the glmmTMB package (Brooks et al., 2017). Due to multi‐collinearity (Pearsons *r* > .7, Figure S3) of the mean, minimum and maximum SST parameters, we used mean SST for all models examining the density of sea urchins and thermal conditions. There was also a negative correlation between latitude and temperature, with temperature decreasing with increasing latitude (*r* > .7, Figure S4). The distributions of *Diadema* spp. and *P. parvispinus* were zero inflated, and required a zero‐inflated Poisson model (Brooks et al., 2017). Assumption and model checks were undertaken visually using Q–Q plots and the spread of residuals was assessed using residuals vs. fitted values. Model significance was tested with Type II Wald Chi‐square Tests using the *Anova* function in the car package (Fox & Weisberg, 2019). Post‐hoc interpretation of models was done using the emmeans package (Lenth, 2024) to back‐transform model coefficients. As temperature and latitude are highly correlated, relationships between density and temperature would also apply to latitude, but for the purpose of this study we used temperature as a predictor of density.

#### 2.5.2 Temporal trends in sea urchin densities

To examine whether species density varied among years we used separate Generalised Linear Mixed Models (GLMM) with a Poisson distribution and a log link function for each species, with year as fixed effect and the random effects of site and transect (transect nested in site) (*n* = 5) in the glmmTMB package (Brooks et al., 2017). The distributions of *Diadema* spp. and *P. parvispinus* were zero inflated, and required a zero‐inflated Poisson model (Brooks et al., 2017). Pairwise post‐hoc comparisons between years were made with Tukey's Tests in the multcomp package (Hothorn et al., 2008). Assumption and model checks were undertaken visually using Q–Q plots and assessing the spread of residuals using residuals vs. fitted values. Model significance was tested with Type II Wald Chi‐square Tests using the *Anova* function in the car package (Fox & Weisberg, 2019).

#### 2.5.3 Temporal patterns in sea urchin population size structure

Size Frequency distributions (SFDs) and pairwise Two Sample Kolmogorov–Smirnov Tests (K‐S tests) were used to compare SFDs of *C. rodgersii, T. australiae*, *Diadema* spp. and *P. parvispinus*. Specifically, SFDs were compared across years to infer temporal trends and were interpreted using descriptive statistics (see below). Pairwise K‐S tests between each combination of year (2010, 2016 and 2019) were used to test for differences in SFDs between years. Using a series of K‐S tests, we determined if SFDs came from the same distribution, or differed from each other (Anderson & Pratchett, 2014; Meesters et al., 2001). The SFDs were used to infer population dynamics including measures of size, skewness, kurtosis, and the coefficient of variation. We used the false discovery rate procedure (Benjamini & Hochberg, 1995; Pike, 2011) to adjust *p*‐values and minimise Type 1 error from multiple comparisons.

## 3 RESULTS

### 3.1 Sea urchin species

We recorded eight sea urchin species on subtropical reefs in the subtropical‐to‐temperate transition zone of east Australia. The most abundant species were the subtropical/temperate species *C. rodgersii* and *T. australiae* (Table 1). The temperate pencil sea urchin *P. parvispinus* and the tropical *Diadema* spp. (*Diadema savignyi* and/or *D. setosum*) were comparatively less abundant. We also recorded the tropical sea urchin *Tripneustes gratilla gratilla* in low numbers in 2019 and the temperate *Heliocidaris* species, *H. erythrogramma* and *H. tuberculata*, at very low densities across all years. The temperate pencil sea urchin *Prionocidaris callista* was rarely observed, with only 5 observations in 2016 (Table 1).

**TABLE 1:** Summary of the descriptive statistics for sample size (*n*), density (m^−2^), Ferets' diameter (mean), coefficient of variation (CV), skewness (*g*_1_) and kurtosis (*g*_2_) for all sea urchin species observed in the biogeographic transition zone of coastal New South Wales, Australia in 2010, 2016 and 2019.

Species	Year	*n*	Density (m^−2^)	Mean	CV	*g*_1 (skew)_	*g*_2 (kurt)_
*Centrostephanus rodgersii*							
	2010	3199	3.18	10.93	0.19	0.16	3.53
	2012	2282	2.61				
	2016	2093	2.46	11.63	0.18	0.315	3.47
	2019	3002	2.92	11.37	0.18	0.34	3.50
*Tripneustes australiae*							
	2010	358	0.35	7.65	0.21	0.94	6.14
	2012	194	0.13				
	2016	21	0.025	9.1	0.15	−0.11	1.95
	2019	18	0.018	8.68	0.16	0.25	4.13
*Diadema* spp. (*D. savignyi or D. setosum*)							
	2010	79	0.079	9.98	0.1	−0.018	3.05
	2012	58	0.066				
	2016	21	0.025	10.81	0.23	−0.28	1.17
	2019	18	0.018	11.08	0.23	0.08	1.72
*Phyllacanthus parvispinus*							
	2010	57	0.057	6.72	0.22	0.42	2.5
	2012	18	0.021				
	2016	43	0.051	6.83	0.17	−0.42	2.71
	2019	35	0.034	6.24	0.18	0.66	2.97
*Tripneustes gratilla gratilla*							
	2010	0		–	–	–	–
	2012	0					
	2016	0		–	–	–	–
	2019	4		–	–	–	–
*Heliocidaris erythrogramma*							
	2010	1		–	–	–	–
	2012	0					
	2016	0		–	–	–	–
	2019	0		–	–	–	–
*Heliocidaris tuberculata*							
	2010	0		–	–	–	–
	2012	0					
	2016	0		–	–	–	–
	2019	1		–	–	–	–
*Phyllacanthus callista*							
	2010	0		–	–	–	–
	2012	0					
	2016	5		–	–	–	–
	2019	0		–	–	–	–

### 3.2 Sea surface temperature (SST)

**FIGURE 2:**
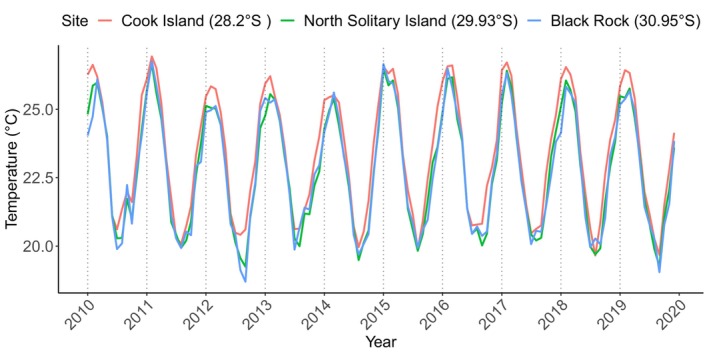
Monthly mean temperature (°C) from the Modis Aqua Satellite Parkinson (2003) for 3 sites (Cook Island, North Solitary Island and Black Rock) in subtropical eastern Australia from 2010 to 2019.

All sites in the region showed strong seasonal variation in temperature (Figures 2 and S2) with the highest range in mean annual temperature from 2010 to 2019 at the most poleward site, Black Rock (18.7–26.76°C), and the lowest range in mean temperature at Julian Rocks (19.46–26.71°C), one of the most northern sites (Figure 1, Table S3). Across the region, mean annual temperature was highest in 2015 (23.14°C) (Table S4) and lowest in 2012 (22.57°C) (Table S4). The lowest minimum average monthly temperature recorded in the study period was in 2012 (18.699°C), at Black Rock and the highest recorded mean monthly temperature in the region was at Cook Island (26.93°C), the most northerly site (Figures 1 and 2). Overall, the site with the highest level of mean annual variation was Cook Island (SEM = 0.2011), and the site with the least variation was South Solitary Island (SEM = 0.194) (Table S3).

### 3.3 Thermal and spatial trends in sea urchin densities

**FIGURE 3:**
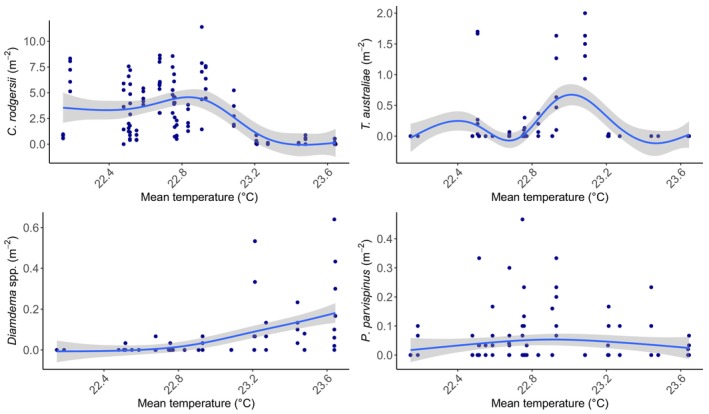
Scatter plots between mean annual temperature (means of monthly means for each year) and the mean density (m^−2^) (per site) of *C. rodgersii, T. australiae, Diadema* spp. and *P. parvispinus* from 2010 to 2019 in the biogeographic transition zone of New South Wales, eastern Australia. Trend lines are General Additive Model predictions between mean temperature and density for each species, the shaded areas show the standard error from the mean.

There was no relationship between the density of *C. rodgersii* (*χ* ^2^ = 0.7978, df = 1, *p* = .37) or *Diadema* spp. (*χ* ^2^ = 0.8039, df = 1, *p* = .3699) and mean temperature in our study region in NSW (Figure 3). The density of *T. australiae* (*χ* ^2^ = 130.19, df = 1, *p* < .001) and *P. parvispinus* varied significantly with mean temperature (*χ* ^2^ = 130.19, df = 1, *p* = .01) (Figure 3) at our study sites in NSW. For every 1°C increase in temperature, *T. australiae* density increased by 0.00126 m^−2^, and *P. parvispinus* density increased by 0.045 m^−2^.

### 3.4 Temporal trends in sea urchin densities and population size structure

#### 3.4.1 Centrostephanus rodgersii

**FIGURE 4:**
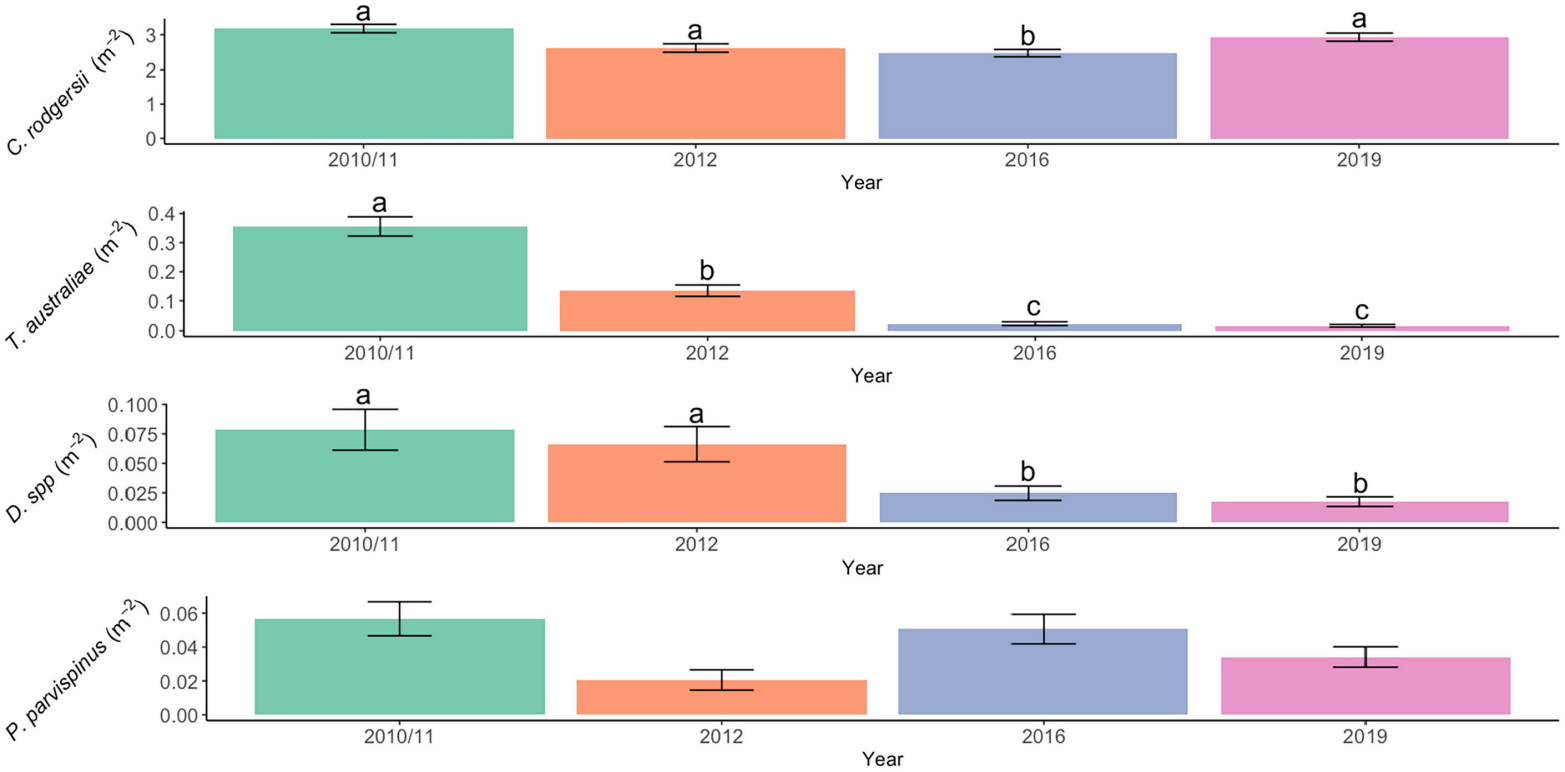
Mean densities (±SEM) of *C. rodgersii*, *T. australiae*, *Diadema* spp. and *P. parvispinus* from 2010 to 2019 in the biogeographic transition zone of New South Wales, eastern Australia. Letters indicate Tukeys post hoc groups. Groups that do not share a letter are significantly different.

**FIGURE 5:**
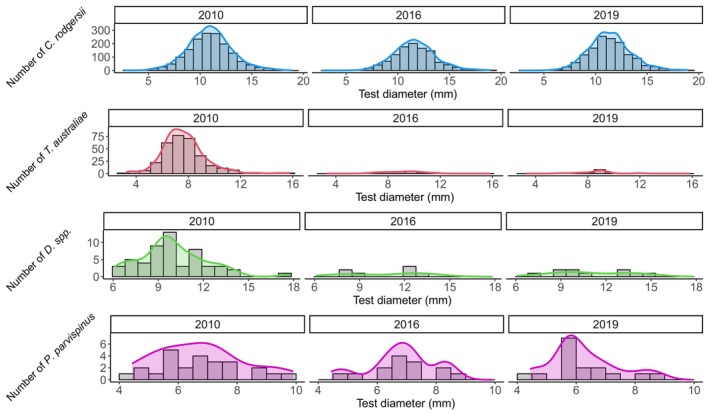
Temporal patterns in the population size structure of *C. rodgersii*, *T. australiae*, *Diadema* spp. and *P. parvispinus* from 2010 to 2019 in the biogeographic transition zone of New South Wales, eastern Australia. Curves overlying the histograms are smoothed density estimates computed using kernel density estimates.

The largest and most abundant sea urchin was *C. rodgersii* (test diameter (TD): 2.7–18.9 cm), with over 4000 individuals measured. Densities of *C. rodgersii* varied through time (*χ* ^2^ = 51.5114, df = 3, *p* < .001) but were the most stable of all the species (Table 1, Figure 4). Size‐frequency distributions of *C. rodgersii* populations differed significantly between years (K‐S test 2010 vs. 2016, *D* = 0.15, *p* = .0015; 2010 vs. 2019, *D* = 0.088, *p* = .0024; 2016 vs. 2019; *D* = 0.07, *p* = .0024). *C. rodgersii* was the largest size in 2016 (Mean TD: 11.63 cm), and the smallest in 2010 (Mean TD: 10.93 cm) (Table 1, Figure 5). However, this species was relatively stable in density and size over the study, as indicated by similar kurtosis, skew and CV values across years (Table 1, Figure 5).

#### 3.4.2 Tripneustes australiae

*T. australiae* was the second most abundant sea urchin species, with over 600 individuals observed. The density of *T. australiae* declined markedly during the study period (*χ* ^2^ = 304.9966, df = 3, *p* < .001) (Table 1, Figure 4). The population size structure of *T. australiae* (TD: 3.3–15.8 cm) significantly differed between 2010 and 2016 (K‐S test: *D* = 0.48, *p* = .0024) and 2010 and 2019 (*D* = 0.51, *p* = .0024) but not between 2016 and 2019 (*D* = 0.3, *p* = .312). Between 2010 and 2016, the *T. australiae* populations declined more than 20‐fold and had a low number of small individuals in 2016, as indicated by negative skew (Table 1). In 2019, some small individuals remained after the population decline and higher kurtosis compared to 2010 and 2016 indicated that there may have been some recruitment (Table 1, Figure 5). However, the abundance of *T. australiae* was significantly lower than in 2010 (Figure 4).

#### 3.4.3 *Diadema* spp.

*Diadema* spp. were the third most common (*n* = 176) and the second largest sea urchin taxon (TD: 6.1–17.8 cm) (Table 1). *Diadema* density varied significantly with time (*χ* ^2^ = 35.2437, df = 3, *p* < .001) and declined throughout the study period (Figure 4). The population density of *Diadema* spp. was the lowest in 2019, suggesting that the population did not recover between 2012 and 2019 (Figure 4). The SFDs of *Diadema* spp. did not differ among years (K‐S test 2010 vs. 2016, *D* = 0.42, *p* = .27; 2010 vs. 2019, *D* = 0.32, *p* = .312; 2016 vs. 2019, *D* = 0.33, *p* = .312) (Figure 5). There was a higher representation of smaller individuals in 2019, as indicated by positive skew suggesting some recruitment (Table 1, Figure 5).

#### 3.4.4 Phyllacanthus parvispinus

*P. parvispinus* was the least abundant species (*n* = 153) (TD: 4.4–9.9 cm), and the density of this species did not vary over time (*χ* ^2^ = 7.0481, df = 1, *p* = .07) (Figure 4), nor did SFDs (K‐S test 2010 vs. 2016; *D* = 0.42, *p* = .312; 2010 vs. 2019; *D* = 0.32, *p* = .62; 2016 vs. 2019; *D* = 0.33, *p* = .62) (Figure 5, Table 1). Given the low abundance of this species in the study region, higher sampling effort may be required to detect changes in the density and population size structure of this species over time.

## 4 DISCUSSION

Across subtropical reefs in the biogeographic transition zone of east Australia, sea urchin density and population size structure varied among species. This result is consistent with observations for other taxa that have exhibited variable population patterns on these subtropical reefs during the study period. For example, in SIMP, there are remarkably stable communities of some fishes and corals (Malcolm & Ferrari, 2019; Mizerek et al., 2021), increases in tropical herbivorous fishes (Smith et al., 2021), declines in kelp (Vergés et al., 2016) and an overall higher diversity of tropical and subtropical fish species than would be expected based on latitude alone (Miller et al., 2023). Our results show that counter to the expectation that cool tolerant/temperate species would decrease and warm affinity/tropical species would increase due to warming in the region, the abundance of tropical *Diadema* spp. and subtropical *T. australiae* declined, while populations of the temperate species *C. rodgersii* were stable.

Over this nine‐year study, *T. australiae* experienced a 20+ fold decrease in numbers. The genus *Tripneustes* exhibits boom‐bust population cycles and experiences die‐offs in heatwave and decreased salinity conditions (Lawrence & Agatsuma, 2020). For example, at subtropical Lord Howe Island, *T. australiae* (then called *Tripneustes gratilla*) established conspicuous aggregations in coral habitat, hypothesised to be driven by anomalous oceanographic events, or favourable effects of predator removal on recruitment and survival of juveniles (Valentine & Edgar, 2010). Valentine and Edgar (2010) note rapid increase of *T. australiae* which changed from rare to >1.3 m^−2^ over a two‐year period at Lord Howe Island, likely due to major sporadic recruitment, subsequently followed by their disappearance. Similarly, abundant populations of *T. australiae* further poleward along the east coast of Australia, in Port Stephens and Sydney in the 1990s (Bové, 2004) did not return over the following 20+ years (Authors, pers. obs). Notably, T. *australiae* recently increased in local abundance in Port Stephens and Sydney in 2023, following what appears to be a similar sporadic recruitment event (Author et al., in prep.).

Along the east Australian biogeographic transition zone, *T. australiae* are living at their warm range‐edges (Author et al., in prep.), and the rapid die off of *T. australiae* followed by population decrease could represent the first two stages of population contraction as described by Bates et al. (2014). Our results show that the abundance of *T. australiae* may be linked to temperature, with increases in density with warmer mean temperature, and a density peak around 23°C. The highest mean temperature and the highest recorded temperature in the study period were in 2011. This may have influenced the population decrease in 2012, suggesting that *T. australiae* may be more sensitive to temperature variation at its warm range edge, especially when compared to the co‐occurring temperate sea urchin *C. rodgersii*.

Species are expected to perform at a lower capacity at their warm range edges (Zarzyczny et al., 2023). As the physiological tolerance limits of *T. australiae* have not been described, and *Tripneustes* are known to experience die‐offs in heatwave conditions and salinity decreases (Lawrence & Agatsuma, 2020), marine heatwaves and storms during our survey period (Lachs et al., 2021) may have contributed to their decline and limited recovery in this region. However, as *T. australiae* sea urchins have been observed in the Solitary Islands region after our study period (Author, pers. obs), the population dynamics of *T. australiae* recorded here appear to reflect a boom‐bust pattern influenced by environmental drivers other than temperature.

*T. australiae* and *Diadema* spp. exhibited dramatic declines across all sizes, which may explain the limited recovery of both species during our study. *Diadema* spp. are living at their cool range edge in this region, likely at the limits of their physiological tolerances and with limited propagules to supply recruitment. This might make them more susceptible to physiological stress and disease (Rowley et al., 2022), and these factors could have contributed to their population decline during the 9‐year period. Although *T. australiae* and *Diadema* spp. populations showed some indications of smaller individuals consistent with recruitment in 2019, their abundances were still much lower than in 2010. Both species are likely to rely on external larval supply to re‐seed their populations in this region. However, it is worth noting that the *Diadema* spp. in this study may have cryptic juveniles and recruits would not be detected by our survey method. In contrast, *T. australiae* are generally in the open, defended by their cover of shells and other items as camouflage and venomous globiferous pedicellariae (Lawrence & Agatsuma, 2020; Sheppard‐Brennand et al., 2017). Thus, we are confident that the smaller size classes of this species would have been captured by our photographic survey method.

The barren forming sea urchin *C. rodgersii* had the most stable population through time, and there was no influence of temperature on population densities which ranged between 2.46 and 3.17 m^−2^ over the study period. All sites included in this study were in Marine Protected Areas (MPAs), and similar stability has been observed in other MPAs in NSW, where *C. rodgersii* populations were stable through time despite increases in urchin predators such as snapper (*Chrysophrys auratus*), eastern blue grouper (*Achoerodus viridis*) and eastern rock lobster (*Sagmariasus verreauxi*) (Glasby & Gibson, 2020, summarised by Przeslawski et al., 2023). In contrast to the range expansion and proliferation of *C. rodgersii* into Tasmania (Ling, 2008), our study highlights that the population dynamics of this sea urchin on subtropical reefs are stable in its historic range in NSW, as noted elsewhere along the NSW coast (Davis et al., 2023; Glasby & Gibson, 2020). In fact, Davis et al. (2023) suggest that *C. rodgersii* populations may begin to decrease in their northern range (including SIMP) with warming. However, we did not see a decline in *C. rodgersii* populations during our study period, which experienced a 0.16°C increase in mean annual mean temperatures between 2003 and 2019. This is consistent with the thermal range of *C. rodgersii* (19.6–26.5°C in its northern distribution (Byrne et al., 2022)) and suggests that *C. rodgersii* is still within its thermal limits in our study region. Other studies have documented declines in kelp (Vergés et al., 2016) and coral populations (Kim et al., 2019; Sommer et al., 2024) in response to marine heat waves in the region (e.g. up to 14 Degree Heating Weeks in 2016; Lachs et al., 2021), and our results suggest that *C. rodgersii* populations in northern NSW were unaffected by these heat stress events.

Our size frequency data suggest that the populations of *C. rodgersii* were stable over the nine‐year study period and that they were dominated by large individuals. This is similar to patterns observed for *C. rodgersii* populations elsewhere in NSW (Andrew & O'Neill, 2000; Andrew & Underwood, 1989). The literature on *C. rodgersii* suggests sporadic recruitment events, with the possibility of density‐dependent recruitment, as the largest observation of juvenile *C. rodgersii* followed mass mortality of adult conspecifics after large storms (Andrew, 1991). *C. rodgersii* juveniles are very cryptic, and rarely observed even in visual census surveys (Andrew & Underwood, 1989). Notably, a recent study observed juvenile *C. rodgersii* emerging at night (Smith et al., 2024) highlighting that night time surveys are required to detect small individuals.

Diadematid sea urchins can facilitate the proliferation of corals on coral and rocky reefs by grazing turfing algae (Lessios, 2016; Lessios et al., 2001; Ling et al., 2018; Sammarco, 1980). The present study revealed sizeable and stable *C. rodgersii* populations in association with corals on subtropical reefs of east Australia. Given the stability of the coral assemblages in the SIMP over 23 years (Mizerek et al., 2021) and the role that sea urchins, particularly diadematids can play in mediating coral success by reducing habitat competition from algae, *C. rodgersii* may mediate the success of corals in these subtropical coral habitats (Lessios, 2016; Lessios et al., 2001; Sammarco, 1980).

This study adds to the growing body of knowledge regarding *C. rodgersii* within its historic range in NSW (Davis et al., 2023; Glasby & Gibson, 2020; Kingsford & Byrne, 2023; Przeslawski et al., 2023) and shows that that *C. rodgersii* populations in this coral associated habitat in NSW are currently stable (Glasby & Gibson, 2020, summarised by Przeslawski et al., 2023). Although the poleward range expansion of *C. rodgersii* has altered the sea scape in Tasmania (Ling, 2008), in NSW, the barrens‐macroalgae mosaic is a natural and stable part of the ecosystem that promotes local biodiversity and is characteristic of this coastal ecosystem (Glasby & Gibson, 2020; Kingsford & Byrne, 2023; Przeslawski et al., 2023). Any management action in our study region, such as culling or expansion of the *C. rodgersii* fishery in NSW, must be carefully considered and informed by research (Kingsford & Byrne, 2023). Further, it should be a research priority to understand the ecological role of the barren forming sea urchin *C. rodgersii* within its preindustrial range, including its interaction with corals and other organisms on subtropical reefs in the biogeographic transition zone.

## 5 CONCLUSION

Subtropical reefs are important ecosystems, and this study is the first to investigate the long‐term temporal dynamics of sea urchin populations in this region, adding to similar long‐term studies of other taxa such as corals, kelps and fishes (Cant et al., 2023; Malcolm & Ferrari, 2019; Sommer et al., 2018; Vergés et al., 2016). Sea urchins are important herbivores, and we show that population dynamics of sea urchins in these subtropical reefs vary among taxa and cannot be predicted by biogeographic affinity alone. Specifically, the most abundant species, the temperate species *C. rodgersii* were stable through time, while the subtropical and tropical species *T. australiae* and *Diadema* spp. declined across all size classes during the 9‐year survey period. Niche availability in a receiving ecosystem is crucial for the establishment of an invading or range extending population (Bates et al., 2014; Miller et al., 2023; Zarzyczny et al., 2023). It is thus possible that subtropical reefs in this region may not experience an influx of tropical sea urchins, while the populations of *C. rodgersii* are dominant and stable, and that *C. rodgersii* mediates ecological dynamics of other taxa in this region.

## AUTHOR CONTRIBUTIONS

**Emily McLaren:** Conceptualization (equal); formal analysis (lead); validation (lead); visualization (lead); writing – original draft (lead); writing – review and editing (lead). **Brigitte Sommer:** Conceptualization (equal); funding acquisition (equal); investigation (lead); methodology (lead); project administration (lead); supervision (equal); writing – review and editing (equal). **Maria Beger:** Funding acquisition (equal); investigation (equal); project administration (supporting); writing – review and editing (equal). **John M. Pandolfi:** Funding acquisition (equal); writing – review and editing (equal). **Maria Byrne:** Supervision (equal); writing – review and editing (equal).

## FUNDING INFORMATION

This research was financially supported by the Australian Government Research Training Program (ATP) PhD scholarship and the Holsworth Wildlife Research Endowment—Equity Trustees Charitable Foundation & the Ecological Society of Australia to EM and an Australian Research Council Discovery Early Career Research Award (DE230100141) to BS. Field work was supported through funding from the Australian Research Council Centre of Excellence for Coral Reef Studies (CE140100020) to JMP and others, a CSIRO Integrated Natural Resource Management Scholarship to BS and the Australian Research Council Centre of Excellence for Environmental Decisions (CE110001014) and the Winifred Violet Scott Charitable Trust to MB.

## CONFLICT OF INTEREST STATEMENT

The authors declare no conflicts of interest.

## Supporting information


**Data S1**.

## Data Availability

Summarised supporting data are available at: https://datadryad.org/stash/share/Br0b0LXpXOQL4TPw2x0n7OtfAgtxdMC3ei5oXWFQLGs. The authors used standard statistical analyses in the freely available software R.
