# Supplementary material for: Taxa‐dependent temporal trends in the abundance and size of sea urchins in subtropical eastern Australia
Source: Ecol Evol. 2024 May 19;14(5):e11412. doi: 10.1002/ece3.11412 (PMC11103280; doi:10.1002/ece3.11412)
Supplement: Supplementary file 1 — Data S1. [file ECE3-14-e11412-s001.docx]

**Supporting information for:**

**Taxa dependent temporal trends in the abundance and size of sea urchins in subtropical eastern Australia**

**Table of Contents:**

| **Supplementary Table S1** | Page 2 |
| --- | --- |
| **Supplementary Figure S1** | Page 3 |
| **Supplementary Figure S2** | Page 4 |
| **Supplementary Table S2** | Page 5-7 |
| **Supplementary Table S3** | Page 8 |
| **Supplementary Table S4** | Page 9 |
| **Supplementary Figure S3.** | Page 10 |
| **Supplementary Figure S4.** | Page 11 |
| **Supplementary Table S5** | Page 12 |

Supplementary Table S1. Species of sea urchins that were observed across the biogeographic transition zone in coastal New South Wales, Australia, in 2010, 2012, 2016 and 2019, and their typical distributional range.

| Species | Range |
| --- | --- |
| *Centrostephanus rodgersii* | Northern New South Wales – Southern Tasmania |
| *Tripneustes australiae* | Northern New South Wales – Southern New South Wales (Montague Island) |
| *Tripneustes gratilla* | Pan-tropical |
| *Diadema* spp*. (Diadema savignyi or D. setosum)* | Pan-tropical |
| *Phyllacanthus parvispinus* | North Queensland – Southern New South Wales |
| *Prionocidaris callista* | North Queensland – Southern New South Wales |
| *Heliocidaris erythrogramma* | Southern Queensland, New South Wales, South Australia, and southern Western Australia |
| *Heliocidaris tuberculata* | Southern Queensland, New South Wales |


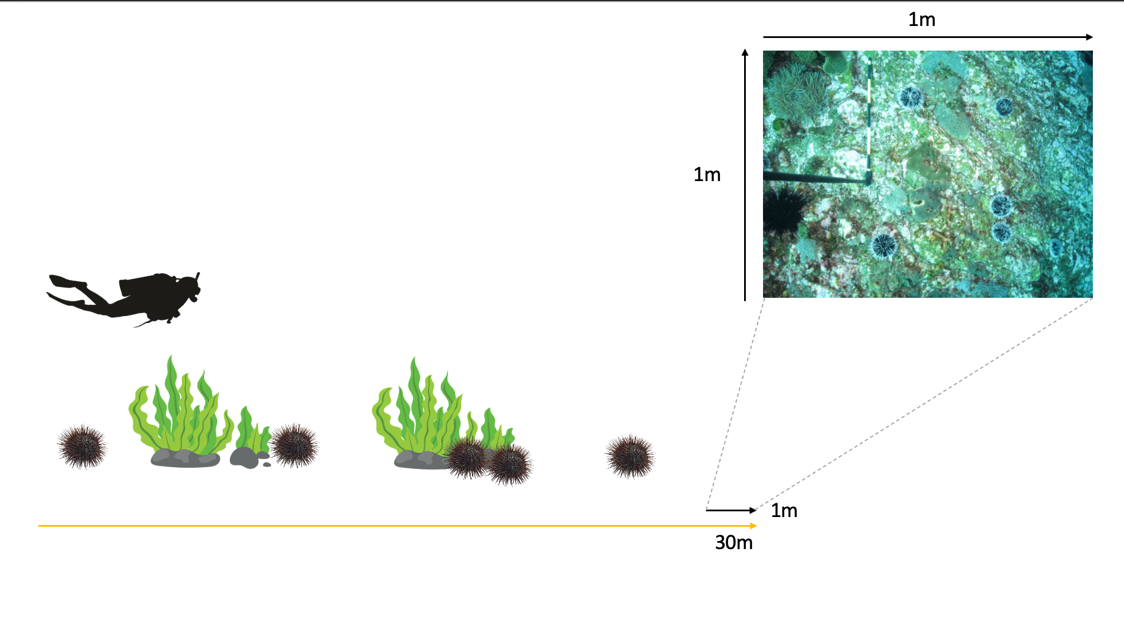


Supplementary Figure S1. Visualisation of the survey method used. Photographs were taken at a consistent depth above the sea floor for a 30m long by 1m wide belt transect between 8 and 12m depth.


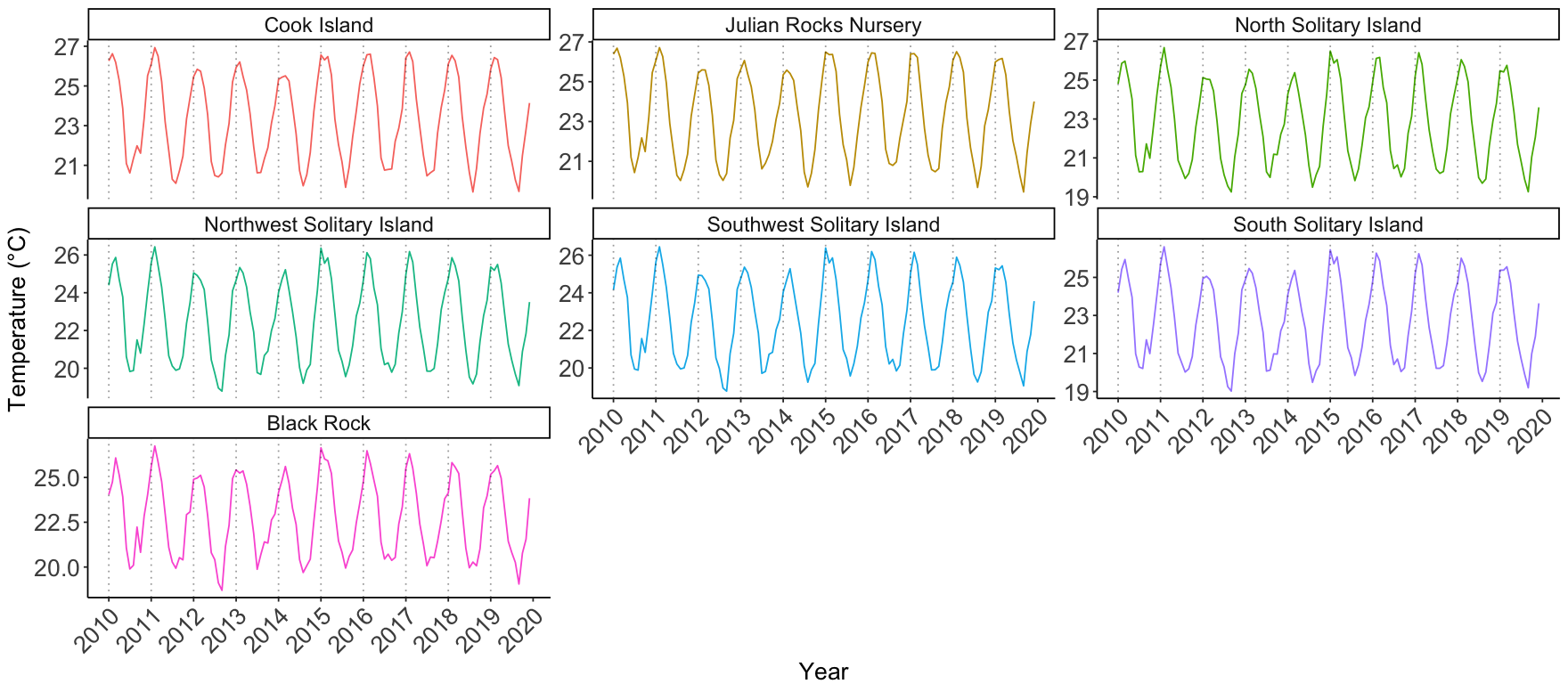


Supplementary Figure S2. Monthly mean sea surface temperature (°C) from the Modis Aqua Satellite (Parkinson 2003) for all subtropical sites of eastern Australia from 2010-2020. Sites arrayed along increasing latitude (28.196°S to 30.95°S) from left to right in each row.

Supplementary Table S2. The mean annual temperature and standard error (°C, ±SEM) for all sites arrayed from low to high-latitude from 2010-2019 (January – December). Red asterisk (*) marks the highest/hottest values and blue asterisk (*) marks the lowest/ coolest.

| **Site** | **Latitude** | **Year** | **Mean temperature (**°C) | **SEM** |
| --- | --- | --- | --- | --- |
| Cook Island | -28.19627,  153.57632 |  |  |  |
|  |  | 2010 | 23.64425* | 0.653457 |
|  |  | 2011 | 23.33375 | 0.717553 |
|  |  | 2012 | 23.212* | 0.632587 |
|  |  | 2013 | 23.29833 | 0.583967 |
|  |  | 2014 | 23.32542 | 0.617113 |
|  |  | 2015 | 23.627 | 0.680988 |
|  |  | 2016 | 23.44108 | 0.663352 |
|  |  | 2017 | 23.44092 | 0.667823 |
|  |  | 2018 | 23.499 | 0.685676 |
|  |  | 2019 | 23.27192 | 0.689328 |
| Julian Rocks Nursery | -28.61087,  153.62809 |  |  |  |
|  |  | 2010 | 23.639* | 0.666457 |
|  |  | 2011 | 23.19042 | 0.706471 |
|  |  | 2012 | 23.08067* | 0.642607 |
|  |  | 2013 | 23.26458 | 0.554666 |
|  |  | 2014 | 23.21442 | 0.635086 |
|  |  | 2015 | 23.52242 | 0.690323 |
|  |  | 2016 | 23.47867 | 0.635684 |
|  |  | 2017 | 23.37217 | 0.666683 |
|  |  | 2018 | 23.45375 | 0.68589 |
|  |  | 2019 | 23.20842 | 0.682087 |
| North Solitary Island | -29.927723, 153.389619 |  |  |  |
|  |  | 2010 | 23.085 | 0.622292 |
|  |  | 2011 | 22.85875 | 0.693013 |
|  |  | 2012 | 22.50717* | 0.653346 |
|  |  | 2013 | 22.76658 | 0.561477 |
|  |  | 2014 | 22.69058 | 0.595378 |
|  |  | 2015 | 23.10117* | 0.681262 |
|  |  | 2016 | 22.8335 | 0.657081 |
|  |  | 2017 | 22.9215 | 0.639734 |
|  |  | 2018 | 22.84067 | 0.667732 |
|  |  | 2019 | 22.75992 | 0.657947 |
| Northwest Solitary Island | -30.018969, 153.269667 |  |  |  |
|  |  | 2010 | 22.771 | 0.636073 |
|  |  | 2011 | 22.624 | 0.692397 |
|  |  | 2012 | 22.14667* | 0.690337 |
|  |  | 2013 | 22.49308 | 0.581324 |
|  |  | 2014 | 22.39067 | 0.613635 |
|  |  | 2015 | 22.82317* | 0.691265 |
|  |  | 2016 | 22.55025 | 0.663087 |
|  |  | 2017 | 22.658 | 0.659485 |
|  |  | 2018 | 22.547 | 0.685761 |
|  |  | 2019 | 22.51392 | 0.662943 |
| Southwest Solitary Island | -30.159215, 153.22809 |  |  |  |
|  |  | 2010 | 22.75667 | 0.616772 |
|  |  | 2011 | 22.66892 | 0.688906 |
|  |  | 2012 | 22.1855* | 0.682118 |
|  |  | 2013 | 22.50708 | 0.583701 |
|  |  | 2014 | 22.41917 | 0.610091 |
|  |  | 2015 | 22.84267* | 0.688252 |
|  |  | 2016 | 22.58758 | 0.661797 |
|  |  | 2017 | 22.67208 | 0.647179 |
|  |  | 2018 | 22.58025 | 0.671596 |
|  |  | 2019 | 22.51542 | 0.661908 |
| South Solitary Island | -30.20478,  153.26515 |  |  |  |
|  |  | 2010 | 22.9305 | 0.59465 |
|  |  | 2011 | 22.82825 | 0.679919 |
|  |  | 2012 | 22.40233* | 0.660952 |
|  |  | 2013 | 22.69692 | 0.565802 |
|  |  | 2014 | 22.6065 | 0.593678 |
|  |  | 2015 | 23.01858* | 0.673844 |
|  |  | 2016 | 22.74858 | 0.651197 |
|  |  | 2017 | 22.84217 | 0.627153 |
|  |  | 2018 | 22.76775 | 0.654827 |
|  |  | 2019 | 22.67825 | 0.653097 |
| Black Rock | -30.948371,  153.076078 |  |  |  |
|  |  | 2010 | 22.90892 | 0.597918 |
|  |  | 2011 | 22.84942 | 0.702599 |
|  |  | 2012 | 22.47933* | 0.694391 |
|  |  | 2013 | 22.90208 | 0.55749 |
|  |  | 2014 | 22.7175 | 0.609029 |
|  |  | 2015 | 23.06642 | 0.691458 |
|  |  | 2016 | 22.92342* | 0.64644 |
|  |  | 2017 | 22.84558 | 0.627427 |
|  |  | 2018 | 22.79383 | 0.641966 |
|  |  | 2019 | 22.67667 | 0.663756 |

Supplementary Table S3. The mean annual mean, minimum and maximum temperatures, standard error (°C, ±SEM), and temperature range for all subtropical sites in eastern Australia from 2010-2019. Black asterisk (*) marks the site with the highest variation in the study period.

| Site | Mean | SEM | Min | Max | Range |
| --- | --- | --- | --- | --- | --- |
| Cook Island | 23.40937 | 0.2011405* | 19.657 | 26.932 | 7.275 |
| Julian Rocks Nursery | 23.34245 | 0.2006128 | 19.461 | 26.713 | 7.252 |
| North Solitary Island | 22.83648 | 0.1964133 | 19.248 | 26.671 | 7.423 |
| Northwest Solitary Island | 22.55177 | 0.2009287 | 18.788 | 26.433 | 7.645 |
| Southwest Solitary Island | 22.57353 | 0.1989232 | 18.765 | 26.448 | 7.683 |
| South Solitary Island | 22.75198 | 0.1941202 | 19.018 | 26.608 | 7.59 |
| Black Rock | 22.81632 | 0.1965385 | 18.699 | 26.756 | 8.057 |

Supplementary Table S4. The mean annual mean, minimum and maximum temperature, standard error (°C, ±SEM), and temperature range for all years (January-December) (across sites). Red asterisk (*) marks the highest/hottest values, blue asterisk (*) marks the lowest/ coolest and black asterisk (*), marks the year with the highest variation.

| Year | Mean | SEM | Min | Max | Range |
| --- | --- | --- | --- | --- | --- |
| 2003 | 22.64585 | 0.2211444 | 18.983 | 25.899 | 6.916 |
| 2004 | 22.57379 | 0.2237762 | 19.45 | 26.481 | 7.031 |
| 2005 | 23.03 | 0.2261151 | 19.756 | 26.463 | 6.707 |
| 2006 | 22.83646 | 0.2215143 | 19.846 | 26.601 | 6.755 |
| 2007 | 22.53848 | 0.2349314 | 18.972 | 25.979 | 7.007 |
| 2008 | 22.41929 | 0.2142782 | 19.083 | 25.578 | 6.495 |
| 2009 | 22.66174 | 0.2301746 | 19.007 | 26.28 | 7.273 |
| 2010 | 23.10505 | 0.2316519 | 19.83 | 26.685 | 6.855 |
| 2011 | 22.90764 | 0.2552618 | 19.893 | 26.932* | 7.039 |
| 2012 | 22.57338* | 0.2459644 | 18.699* | 25.841 | 7.142 |
| 2013 | 22.84695 | 0.2101528 | 19.676 | 26.209 | 6.533 |
| 2014 | 22.76632 | 0.2254355 | 19.203 | 25.613 | 6.41 |
| 2015 | 23.14306* | 0.2515467* | 19.557 | 26.654 | 7.097 |
| 2016 | 22.93758 | 0.2412529 | 19.791 | 26.603 | 6.812 |
| 2017 | 22.96463 | 0.238139 | 19.845 | 26.713 | 6.868 |
| 2018 | 22.92604 | 0.2473801 | 19.173 | 26.543 | 7.37 |
| 2019 | 22.8035 | 0.2450272 | 19.046 | 26.431 | 7.385 |


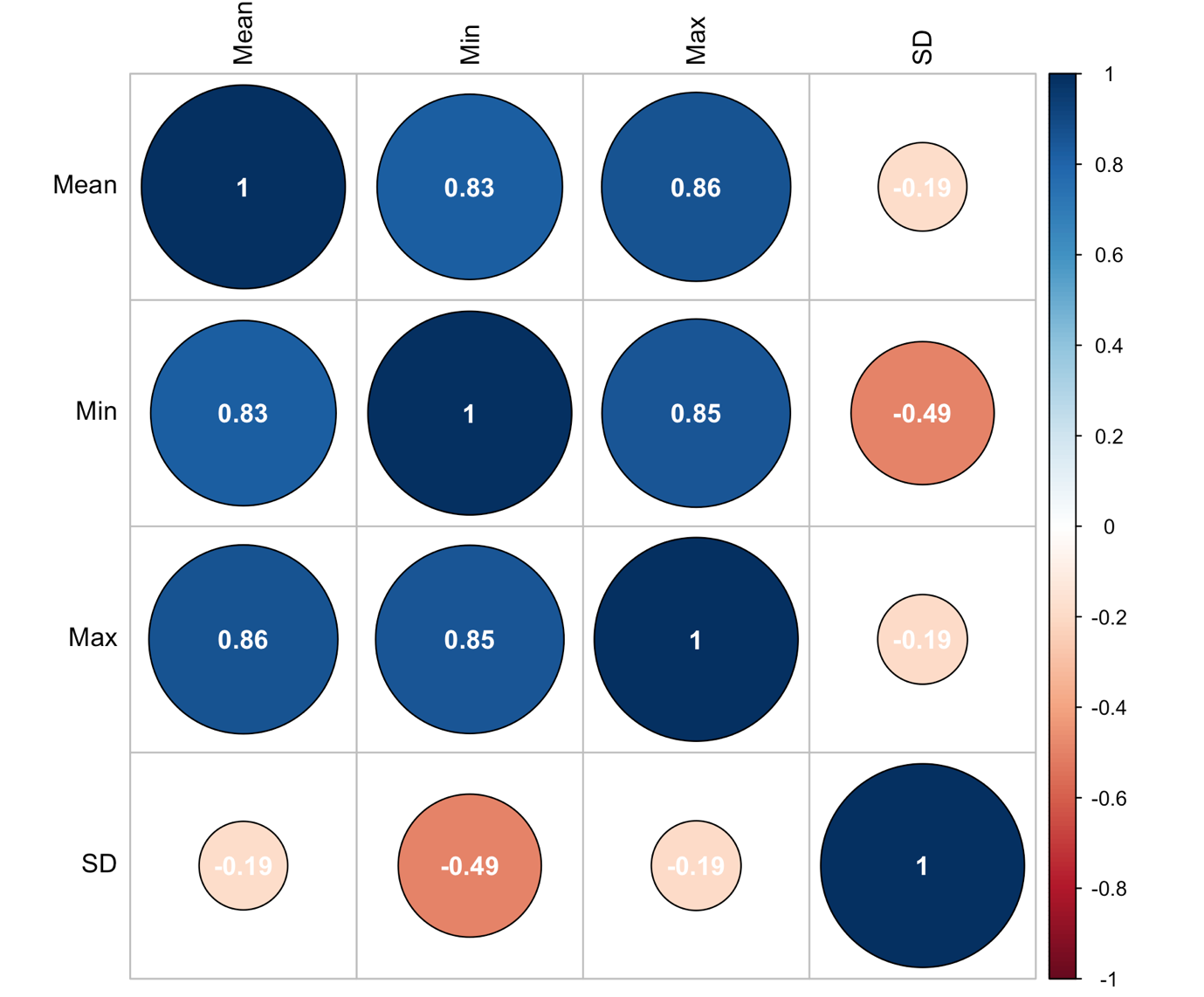


Supplementary Figure S3. Correlation matrix of temperature parameters extracted for all sites from the Modis Aqua satellite (Parkinson 2003) at 1km spatial resolution for 2010-2019.


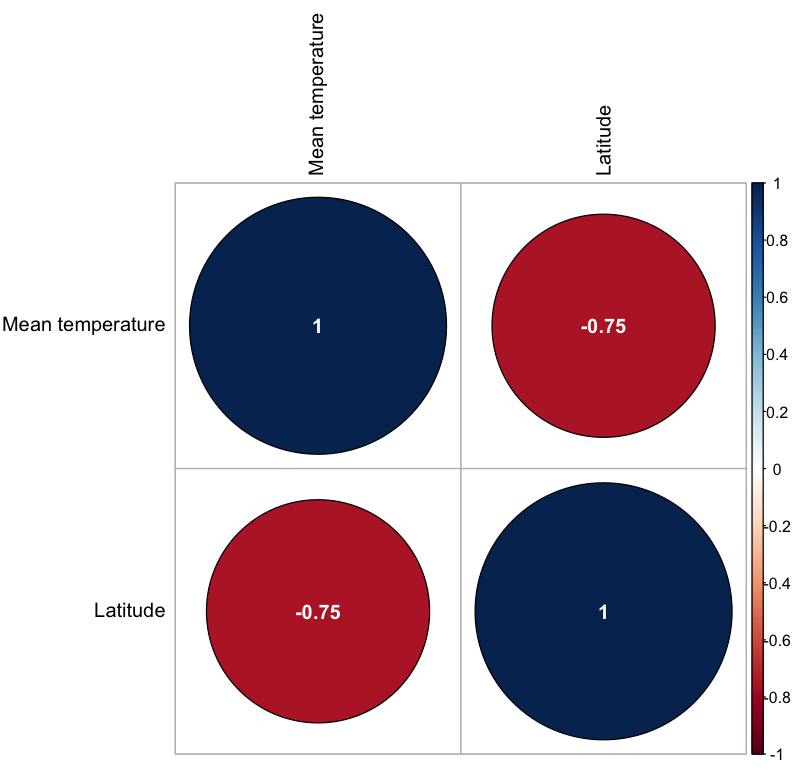


Supplementary Figure S4. Correlation matrix of mean annual temperature and latitude for all sites from the Modis Aqua satellite (Parkinson 2003) at 1km spatial resolution for 2010-2019.

Supplementary Table S5. Table of GLMM results for the models examining the relationship between mean temperature and sea urchin densities, and year and sea urchin densities.

| Model |  | χ^2^ | df | p |
| --- | --- | --- | --- | --- |
| Mean temperature ~ species density (m^-2^) |  |  |  |  |
|  | *C. rodgersii* | 0.7978 | 1 | 0.37 |
|  | *Diadema* spp. | 0.8039 | 1 | 0.3699 |
|  | *T. australiae* | 130.19 | 1 | <0.001* |
|  | *P. parvispinus* | 130.19 | 1 | 0.01* |
|  |  |  |  |  |
| Year ~ species density (m^-2^) |  |  |  |  |
|  | *C. rodgersii* | 51.5114 | 3 | <0.001* |
|  | *Diadema* spp. | 35.2437 | 3 | <0.001* |
|  | *T. australiae* | 304.9966 | 3 | <0.001* |
|  | *P. parvispinus* | 7.0481 | 3 | 0.07 |
